# Effect of prophylactic anti-VEGF injections on the prevention of recurrent vitreous hemorrhage in PDR patients after PRP

**DOI:** 10.1038/s41598-022-17214-y

**Published:** 2022-08-25

**Authors:** Jae Wan Lim, Sang Joon Lee, Jae Yun Sung, Jin-soo Kim, Ki Yup Nam

**Affiliations:** 1 Department of Ophthalmology, College of Medicine, Kosin University, Busan, South Korea; 2 Department of Ophthalmology, College of Medicine, Chungnam National University, Daejeon, South Korea; 3 Department of Ophthalmologythalmology, Chungnam National University Sejong Hospital, 20, Bodum 7-ro, Sejong, 30099 South Korea

**Keywords:** Eye diseases, Diabetes complications

## Abstract

We evaluated the effectiveness of intravitreal anti-vascular endothelial growth factor (anti-VEGF) antibody injection (IVAI) for the prevention of recurrent vitreous hemorrhage (VH) due to neovascularization on disc (NVD) in patients with proliferative diabetic retinopathy (PDR) after panretinal photocoagulation (PRP). This retrospective case series reviewed the medical records of 12 PDR patients with recurrent VH after PRP from NVD. The interval between IVAIs was decided on the basis of the interval between VH recurrences after the initial IVAI, and NVD regression/recurrence during follow-up. We recorded the success rate of VH prevention, and the interval between IVAIs. Fundus examination revealed NVD regression at 1 month after the injection. However, NVD progressed gradually and VH recurred after 3–4 months. Thereafter, IVAIs were administered every 3–4 months; VH did not recur and visual acuity remained stable during the treatment period. In one case, NVD did not recur after 4 years of periodic injections. No systemic or ocular complications of IVAI were observed. In conclusion, proactive and periodic IVAIs (at 3–4-month intervals) may prevent recurrent VH in association with NVD in PDR patients after PRP.

## Introduction

Approximately 32–39% of proliferative diabetic retinopathy (PDR) patients develop vitreous hemorrhage (VH) after panretinal photocoagulation (PRP)^1–3^. Severe non-clearing VH requires surgical treatment, whereas less severe cases can be managed conservatively with observation, head elevation, and repeat laser treatment for remnant ischemic areas after the initial laser treatment. Focal laser treatment may be considered for VH due to neovascularization elsewhere (NVE)^4^.

Recently, several studies have reported the effectiveness of intravitreal anti-vascular endothelial growth factor (anti-VEGF) antibody injection (IVAI) compared to PRP as a treatment for PDR patients^5–7^. Also, a few previous studies have reported that IVAI is effective for the treatment of VH in PDR patients^8,9^.

VH may develop after PRP in PDR patients due to persistent neovascularization on disc (NVD). Such patients can be treated with IVAIs^8,9^. However, repeated IVAIs are necessary because the drug effect is temporary and VH may recur. We experienced some patients with recurrence of VH at relatively periodic intervals. Therefore, we believe that if the timing of VH recurrence can be predicted, it can be prevented by IVAI before that.

In the present study, we evaluated whether proactive and repeated IVAIs in these patients can prevent recurrent VH.

## Methods

The protocol of this retrospective case series was approved by the Institutional Review Board of Kosin University Gospel Hospital. The study was performed in accordance with the Declaration of Helsinki. The requirement for informed consent was waived by the Institutional Review Board due to the retrospective study design.

We reviewed the medical records of 12 PDR patients who developed recurrent VH after PRP due to NVD and were treated with periodic IVAIs before recurrences. All patients underwent fundus photography (Kowa Nonmyd 7; Kowa Co. Ltd., Nagoya, Japan/Optos Daytona, Dunfermline, UK), optical coherence tomography (HRA Spectralis + OCT, Heidelberg Engineering, Heidelberg, Germany), and fluorescein angiography (FA; HRA Spectralis, Heidelberg Engineering, Heidelberg, Germany) at initial visit.

When VH first recurred after PRP, fluorescein angiography (FA) was performed to identify the position of new vessels. NVD without NVE was treated with intravitreal bevacizumab injection, followed by laser treatment to the area with insufficient PRP according to FA. The patients were followed monthly to determine the interval between VH recurrences; then IVAIs were performed at regular interval, before the expected timing of recurrence.

Age, sex, visual acuity, follow-up duration, recurrence interval, and total number of injections were recorded by reviewing the medical records.

## Results

### Baseline characteristics

The study participants included eight males and four females, and their mean age was 46.8 (± 12.9) (27–68 years). Five and seven patients were pseudophakic and phakic, respectively. The mean values of the initial and final visual acuity (Logarithm of the Minimum Angle of Resolution) were 0.63 (± 0.43) and 0.09 (± 0.12), respectively. The average follow-up period was 31.6 (± 9.8) months (24–60 months) (Table 1).

**Table 1:** The characteristics of the patients.

Case No	Age (years)	Sex	Lens status	Initial VA (LogMAR)	Final VA (LogMAR)	Follow up duration (months)	Duration to 1^st^ VH recurrence (months)	Recurrence interval	Total injection No. (months)	Average injection No. per year
1	39	F	Phakic	0.2	−0.07	60	8	4	9	2.1
2	41	M	Phakic	0.3	0.09	32	6	4	6	2.8
3	51	M	Phakic	0.39	0.22	24	4	3	5	3
4	58	M	Pseudophakic	0.69	0.04	28	6	3	7	3.8
5	68	F	Pseudophakic	1.0	0.3	36	5	4	7	2.7
6	33	M	Phakic	1.0	0	24	6	4	6	4
7	45	M	Phakic	0.69	0.15	32	4	4	7	3
8	31	M	Phakic	0.52	0.09	26	4	3	6	3.1
9	27	F	Phakic	0.39	0.09	28	7	3	6	3.4
10	62	M	Pseudophakic	0.3	0	26	5	4	5	2.9
11	56	F	Pseudophakic	0.39	−0.07	35	6	4	7	2.9
12	50	M	Pseudophakic	1.69	0.22	28	5	3	6	3.1
Mean (± SD)	46.8 (± 12.9)			0.63 (± 0.43)	0.09 (± 0.12)	31.6 (± 9.8)	5.5 (± 1.2)			3.1 (± 0.5)

*No.* number, *LogMAR* Logarithm of the Minimum Angle of Resolution, *VH* vitreous hemorrhage, *F* female, *M* male, *SD* standard deviation.

### Representative case

**Figure 1:**
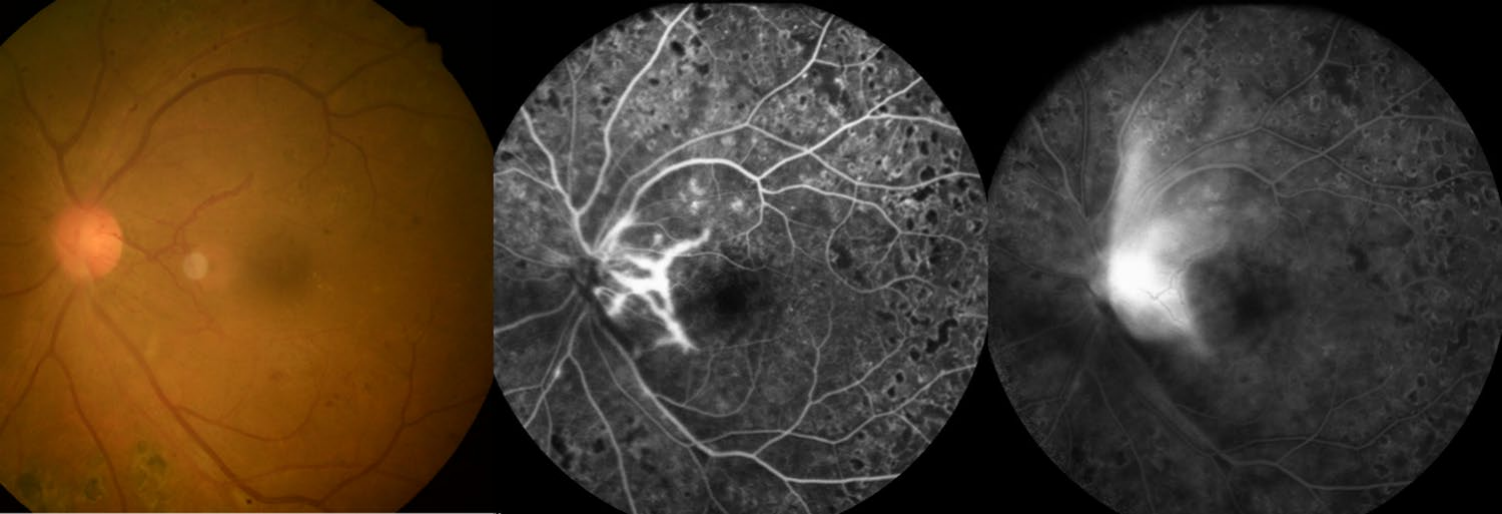
Fundus photographs and fluorescein angiography of case 1 at 6 months after PRP. Engorged new vessel on disc and severe leakage therefrom are observed.

**Figure 2:**
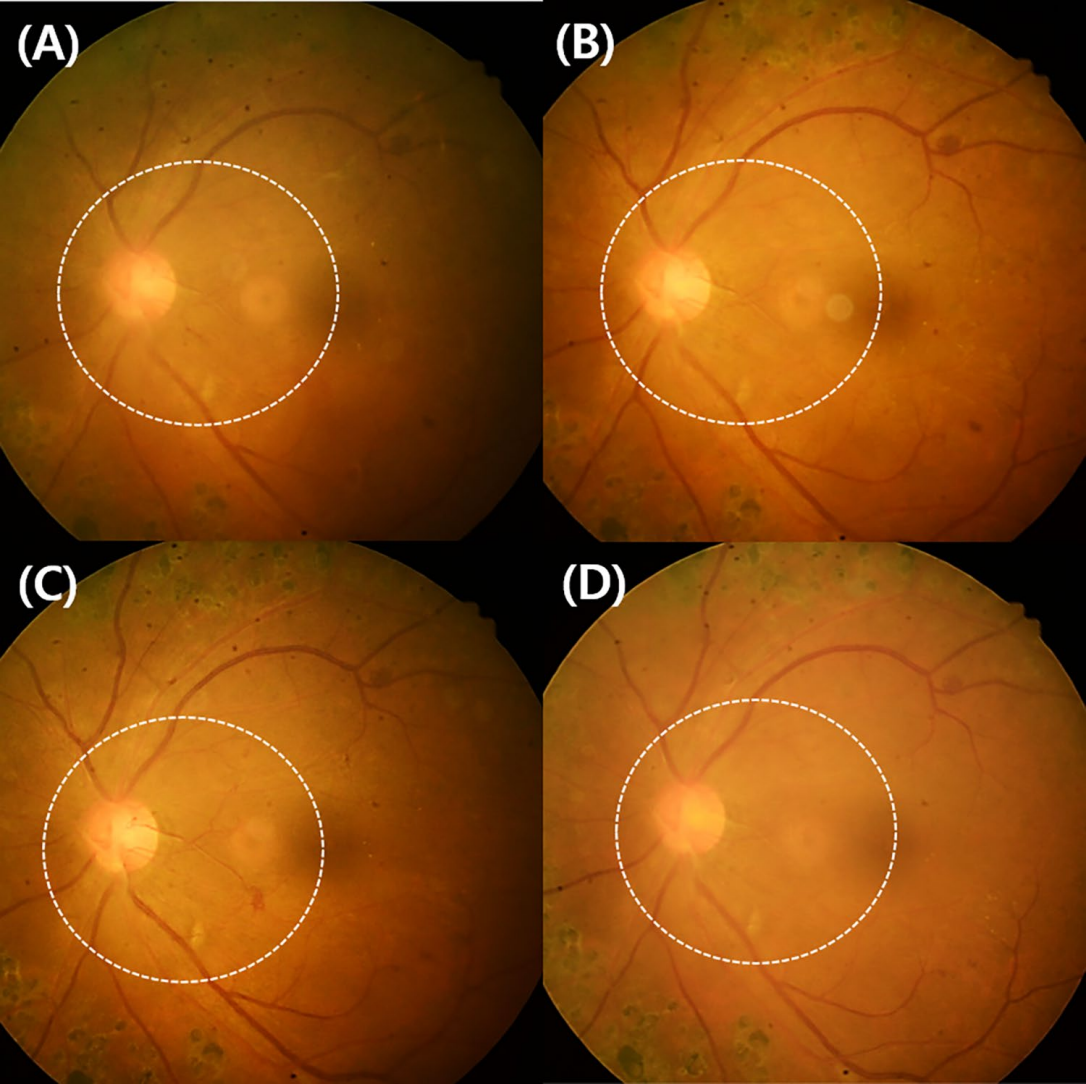
Change in the new vessel on the disc of case 1 before and after anti-vascular endothelial growth factor antibody injection (IVAI) treatment: (**A**) 1 month after the initial IVAI, (**B**) 2 months after the initial IVAI, (**C**) 3 months after the initial IVAI, and (**D**) 1 month after the second IVAI. NVD regressed immediately after IVAI, but progressed over time and was maximally engorged 4 months after IVAI.

**Figure 3:**
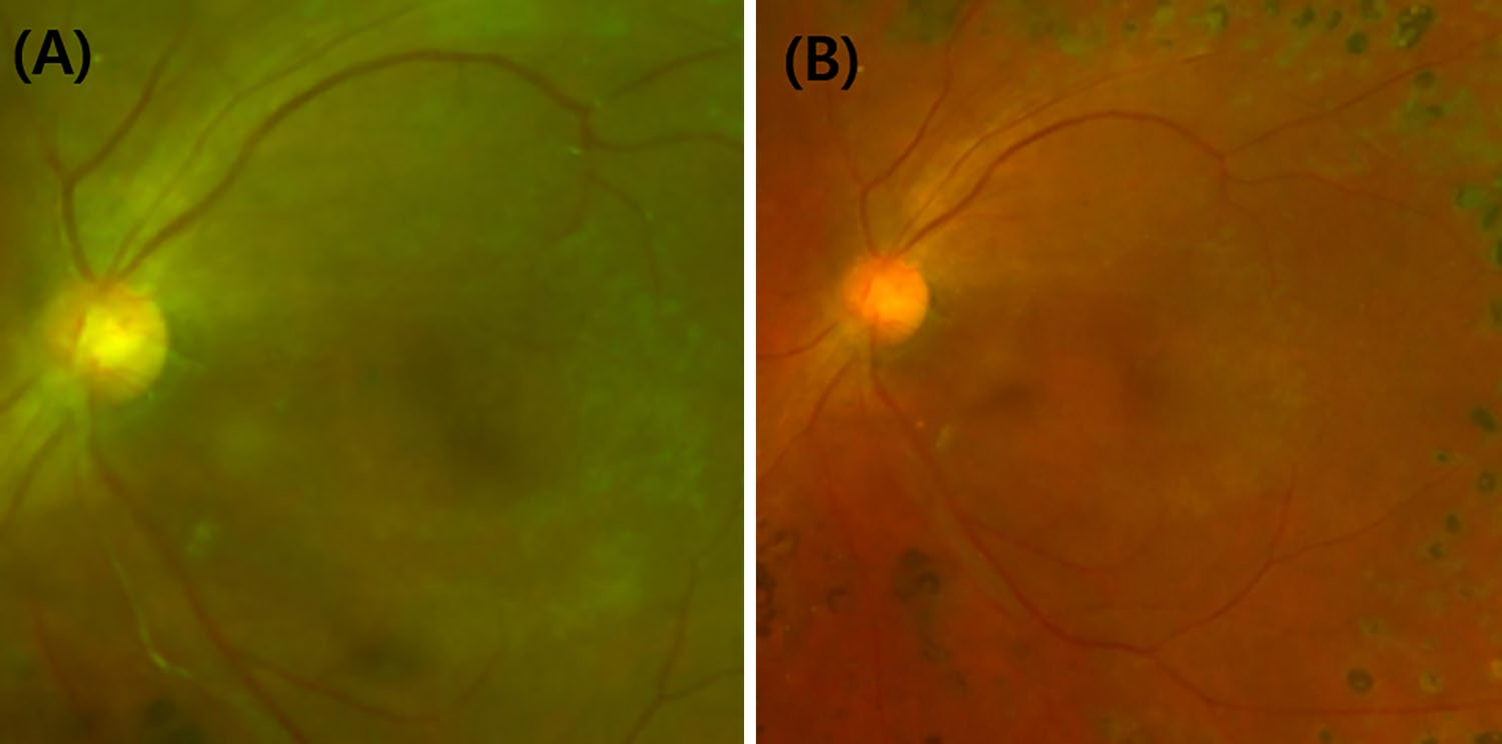
Fundus photographs of case 1 at (**A**) 6 months and (**B**) 18 months after the final IVAI. NVD regeneration and VH recurrence were not observed during the 18-month follow-up.

The first patient a 39-year-old female followed up for 6 years. She initially presented to the clinic with a left eye floater and best-corrected visual acuity of 1.0 and 0.63 in the right and left eyes, respectively. She said she had no history of diabetes mellitus. Fundus examination showed mild VH in left eye and new vessels in both eyes. FA revealed bilateral NVE and NVD. Her HbA1c level was 10.1%, for which she was referred to the endocrinology department. We performed PRP in both eyes. Six months thereafter, NVD progressed in her left eye. Repeat FA revealed severe NVD leakage without NVE, and additional laser treatment was performed in areas not adequately treated with PRP (Fig. 1). However, NVD was not reduced, and moderate VH occurred almost 2 months after the additional laser treatment. As a result, IVAI was performed, which led to improvements in VH and NVD. However, NVD reappeared over time (Fig. 2) and VH recurred 4.5 months after the initial IVAI. Therefore, the patient was monitored for NVD and IVAI was repeated at 4-month intervals. The patient underwent nine IVAIs over 52 months, and had no NVD regeneration or VH recurrence during 18 months after last treatment. (Fig. 3).

### Prophylactic IVAI for recurrent VH

Based on our experience with the above patient, we performed prophylactic IVAI to 11 similar patients at regular intervals. The initial VH occurred 4–8 (mean: 5.8 ± 1.5) months after PRP completion. The patients were treated with IVAI with or without additional laser treatment. The interval between IVAIs was determined based on NVD changes and the duration between the initial IVAI and first VH recurrence.

The interval between VH recurrences was 3–4 months. None of the patients experienced VH recurrence during the follow-up. The average total number of IVAIs per year was 3.1 (± 0.5).

**Figure 4:**
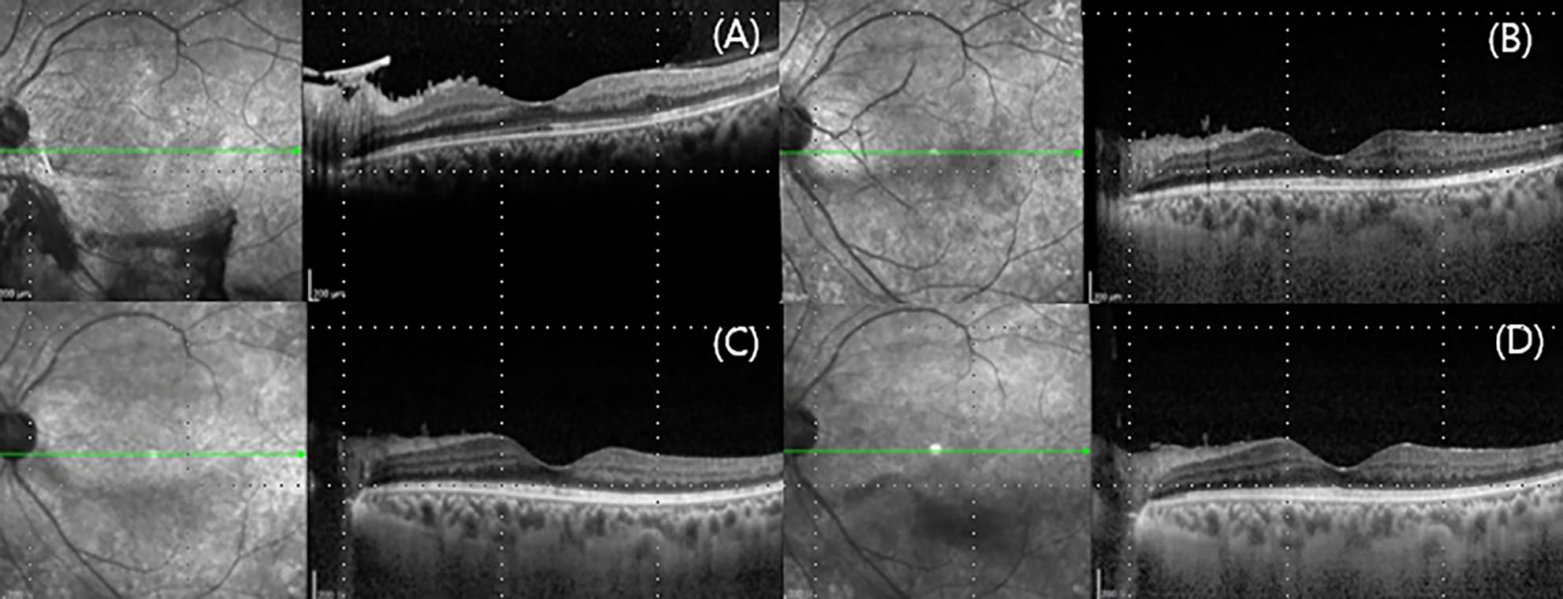
Optical coherence tomography of case 1 at (**A**) initial visit, (**B**) 24 months, (**C**) 48 months, and final visit. No diabetic macular edema developed during the follow-up period.

NVD changes before and after IVAIs were noted. NVD regressed after IVAI but recurred over time. IVAI was performed before VH recurrence when the NVD was maximal. During the follow-up period, the visual acuity remained stable. None of the patients developed macular edema or traction retinal detachment, and none required vitrectomy (Fig. 4). No systemic or ocular complications of IVAI were noted.

## Discussion

We performed prophylactic IVAIs in PDR patients for the prevention of recurrent VH due to NVD. None of the patients experienced VH recurrence during IVAI treatment at 3–4-month intervals.

Several studies have reported that IVAI is effective for the treatment of VH in PDR patients. Wirkkala et al.^9^ reported that ranibizumab injection improved VH clearance compared to other treatments, such as PRP, and observation. The interval from treatment to VH clearance was shorter, and the proportion of patients with VH resolution at 3 months was significantly higher, after ranibizumab treatment compared to the other treatments. In addition, recurrences were significantly less common after ranibizumab compared to the other treatments.

Sinawat et al.^8^ reported that intravitreal bevacizumab injection was effective for the treatment of dense VH after PRP, and was associated with VH clearance in 72.2% of patients over 12 months. However, VH recurred in 56% of patients with a history of PRP after intravitreal bevacizumab injection.

Previous studies have focused on the effect of IVAI on VH improvement rather than VH prevention. Although treatment after VH occurrence may be meaningful, but until the VH is absorbed, the patient experiences discomfort from vision loss. The discomfort is more severe in patients with only one eye functioning. In our study, 4 of 12 patients had poor vision in the opposite eye because of foveal damage related to diabetic retinopathy. These four patients could avoid the discomfort from repeated VH through prophylactic IVAIs.

To date, no studies have investigated prophylactic treatment of VH in PDR patients. VH occurred in more than one-third of patients after PRP^1–3^. Additional laser treatment can be performed in peripheral non-perfused areas after initial laser therapy. Direct coagulation of NVE may also be effective. However, laser treatment is not appropriate for NVD and performed IVAIs for VH treatment. Initially, the VH improved and NVD regressed, but VH recurred and NVD progressed almost 3–4 months after IVAI. Therefore, we assumed that prophylactic IVAIs could prevent VH recurrence if performed according to a regular schedule.

Jorge et al.^10^ investigated NVD changes after IVAI using FA, and found that NVD leakage declined within 24 h after intravitreal bevacizumab injection; moreover, the reduction was maintained until week 6. NVD leakage recurred in 93% of patients at 12 weeks after treatment. In the 5-year extension study of Protocol S, ranibizumab injections were repeated in cases of neovascular worsening, the average number of injections per year to almost 3^6^, i.e., the interval between injections was almost 4 months. The previous studies showed similar results in terms of the interval between VH recurrences and NVD change in the present study.

One of the things to consider in proactive treatment is how long the treatment should be maintained. Our experience with one of the patients suggested that regular IVAI treatment can be discontinued. In one patient who was followed for 5 years, NVD did not progress and VH did not recur until 18 months after the final IVAI. As a result, the treatment discontinuation may be decided on the basis of NVD change.

Although vitrectomy may be performed to treat VH, it carries certain risks in PDR patients. In comparison, IVAI is simple and less invasive than vitrectomy. Repeated IVAIs may also be associated with complications, including endophthalmitis; however, the incidence of endophthalmitis is low. In the 5-year extension study of Protocol S, only one patient developed endophthalmitis among 4,113 injections in 306 eyes^6^. In addition, the average number of annual IVAIs was three, which is relatively low considering the treatment of age-related macular degeneration. Nevertheless, it is necessary to evaluate the risk of endophthalmitis after repeated IVAIs, particularly in patients with a single functioning eye. In addition, the efficacy and safety of IVAIs should be investigated in prospective studies with large sample sizes by comparing prophylactic IVAIs with other treatments, such as early vitrectomy.

This study had several limitations. First, it used a retrospective design and had a small sample size, which makes it difficult to generalize our results. However, our results are meaningful because they suggest the feasibility of a less invasive treatment option than surgery for VH. A prospective study with more patients is needed to confirm our results.

In conclusion, an IVAI schedule based on the VH recurrence interval and NVD change prevents recurrent VH due to NVD even after complete PRP. In the present study, the treatment interval was 3–4 months. Prophylactic IVAIs may be an option to prevent VH recurrence and avoid the need for surgery. NVD change is an important indicator of IVAI treatment discontinuation.

## Data Availability

All of the datasets are presented in the main manuscript.
